# Transarterial chemoembolisation (TACE) using irinotecan-loaded beads for the treatment of unresectable metastases to the liver in patients with colorectal cancer: an interim report

**DOI:** 10.1186/1477-7819-7-80

**Published:** 2009-11-03

**Authors:** Robert CG Martin, Ken Robbins, Dana Tomalty, Ryan O'Hara, Petar Bosnjakovic, Radek Padr, Miloslav Rocek, Frantisek Slauf, Alexander Scupchenko, Cliff Tatum

**Affiliations:** 1 University of Louisville School of Medicine, Division of Surgical Oncology, Louisville, USA; 2 Baptist Health, Little Rock, Arkansas, USA; 3 Huntsville Hospital, Huntsville, Alabama, USA; 4 Radiology & Imaging Consultants, PC, Institute for Minimally Invasive Therapy, Colorado Springs, CO, USA; 5 Institute of radiology Clinical center Nis, Serbia; 6 Departments of Radiology, Pediatric Hematology and Oncology, University Hospital Motol and 2nd Medical Faculty, Charles University, Prague, Czech Republic; 7 Institute of Clinical and Interventional Radiology (IKEM), Department of Diagnostic and Interventional Radiology, Videnska 800, 14000 Prague 4, Czech Republic; 8 Regional Oncological Dispenser, Samara, Russia; 9 Norton Radiology, Louisville, Ky, USA

## Abstract

**Background:**

Following failure of standard systemic chemotherapy, the role of hepatic transarterial therapy for colorectal hepatic metastasis continues to evolve as the experience with this technique matures. The aim of this study to gain a better understanding of the value of drug eluting bead therapy when administered to patients with unresectable colorectal hepatic metastasis.

**Methods:**

This was an open-label, multi-center, single arm study, of unresectable colorectal hepatic metastasis patients who had failed standard therapy from 10/2006-10/2008. Patients received repeat embolizations with Irinotecan loaded beads(max 100 mg per embolization) per treating physician's discretion.

**Results:**

Fifty-five patients underwent 99 treatments using Irinotecan drug eluting beads. The median number of total treatments per patient was 2(range of 1-5). Median length of hospital stay was 23 hours(range 23 hours - 10 days). There were 30(30%) sessions associated with adverse reactions during or after the treatment. The median disease free and overall survival from the time of first treatment was 247 days and 343 days. Six patients(10%) were downstaged from their original disease status. Of these, four were treated with surgery and two with RFA.

Neither number of liver lesions, size of liver lesions or extent of liver replacement(<= 25% vs >25%) were predictors of overall survival. Only the presence of extrahepatic disease(p = 0,001), extent of prior chemotherapy (failed 1^st ^and 2^nd ^line vs > 2 line failure)(p = 0,007) were predictors of overall survival in multivariate analysis.

**Conclusion:**

Chemoembolization using Irinotecan loaded beads was safe and effective in the treatment of patients as demonstrated by a minimal complication rate and acceptable tumor response.

## Background

Surgical resection of the affected portion of the liver offers the best chance for disease-free and overall survival in patients with colorectal hepatic metastasis (CRHM)[1,2]. Unfortunately, most patients present with disease that is not amenable to resection or have other contraindications to surgery. As a result of these limitations, it is estimated that only 15-30% of patients are suitable surgical candidates at initial presentation.

Following standard systemic chemotherapy, additional therapies include transarterial chemotherapy, ethanol injection, cryotherapy, radiofrequency ablation, and microwave ablation. The role of hepatic transarterial therapy for CRHM continues to evolve as experience with this technique matures[3]. There have been recent reports of precision transarterial therapy in metastatic colorectal cancer with acceptable results[4,5]. Chemoembolization offers the promise of even more effective control by combining tumor embolization with prolonged and locally enhanced chemotherapy[6,7]. CRHM are well suited for chemoembolization through the arterial route, since they have a predominantly arterial blood supply[8,9], and most are hypervascularized[10]. Chemoembolization of liver malignancies, including CRHM, have been reported since 1981[11].

A new drug eluting bead treatment represents a new but clinically unproven delivery device that can deposit a chemotherapeutic agent in the liver with minimal release into adjacent tissues[12]. The agent is embedded in beads enough to minimize diffusion by embolizing the terminal capillaries[13]. Modern angiographic techniques can deliver these beads directly to the tumor without imposing an undue risk[5]. The objective of treatment with drug eluting beads is to selectively administer a potentially lethal dose of chemotherapeutic material to the liver metastises while minimizing systemic side effects.

Recent reports from Alberti *et al*, and Fiorentini *et al*, have shown that this drug eluting therapy is generally well-tolerated by patients[4,5]. Major risks include liver failure and gastric irritation caused by seepage into the gastrointestinal tract. Until now, the effectiveness of this device for the treatment of CRHM has not been examined in a large-scale study or in a multi-institutional trial. We have recently published our initial pilot safety data demonstrating this device to be safe in the treatment of metastatic colorectal cancer[14].

The goals of this analysis was to: 1) gain a better understanding of the value of drug eluting bead therapy when administered to patients with unresectable vascular tumors of the liver. 2) Assess the limitations, concerns, and complications that earlier users of drug eluting bead therapy have encountered. This is our interim report of those cases with unresectable liver metastases from colorectal cancer that have been treated with the Irinotecan drug eluting bead (DEBIRI).

## Methods

From January 2007 to October 2008, we conducted a prospective, multi-institutional registry of 55 patients with liver dominant metastatic colon cancer (MCC). Table 1 shows the participating sites in the US, Canada, Europe and Australia. This registry was non-controlled, but it received an IRB approval and complied with the protocol and principles laid down in the Declaration of Helsinki, in accordance with the ICH Harmonized Tripartite Guideline for Good Clinical Practice (GCP). The following criteria were strictly observed: 1) The patient population was well described; 2) The data were carefully obtained; 3) Outcomes were independently assessed; 4) Follow up information was clinically relevant, and few patients were lost to follow up; 5) Comparable patient information was obtained at all the participating institutions[15].

**Table 1:** Number of patients enrolled at each site.

**Site**	**Country**	**Number of patients enrolled**
University of Louisville	US	21

Baptist Health, Little Rock, AR	US	11

Colorado Springs	US	3

Huntsville, AL	US	10

Midland Memorial Hospital, TX	US	1

Centar Nis Serbia	Serbia	2

Usti Nad Labem	Czech Republic	1

Regional Hospital Novy Jicin	Czech Rebublic	1

FN v Motole	Czech Republic	2

FH Plzen	Czech Republic	1

Regional Oncological Dispenser, Samara	Russia	2

Each potential subject was given ample time to decide whether to participate in the study and was informed that they could withdraw at any time.

Inclusion criteria for chemoembolization were: 1) A confirmed diagnosis of liver dominant metastatic colorectal cancer (by either a liver biopsy on past history of colon cancer); 2) An ECOG Performance Status Score of 0 to 2 or a Karnofsky's Performance score of 60 to 100%; 3) Age 18 years or older; 4) Patient able to comprehend the nature of the study and provide informed consent in accordance with institutional and national guidelines. Exclusion criteria were: 1) History of severe allergy or intolerance to any contrast media not controlled with pre-medication; 2) Bleeding diathesis, not correctable by the usual forms of therapy; 3) Severe peripheral vascular disease that would preclude catheterization; 4) Significant extra-hepatic disease, generally in excess of 50% of the overall whole body tumor bulk outside the liver, or any tumor burden that represented an imminent threat to the patient's life; 5) Greater than 75% hepatic parenchymal involvement; 6) Severe liver dysfunction; 6) An active, uncontrolled infection.

Treatment was performed in an outpatient setting via a lobar approach, based on the extent and distribution of the disease. The method of DC/LC Bead therapy has been described previously[14].

The drug eluting bead (DEBIRI) utilized in this report is the DC/LC Bead™ (Biocompatibles, Farnham, UK), which is a PVA microsphere with FDA clearance as a Class II device. It is also CE marked as a Drug Delivery Embolization System. In this study, the DC/LC Bead was loaded with irinotecan in an off label use. DC/LC Bead is available in the size ranges of 100 - 300 μm, 300 - 500 μm, 500 - 700 μm and 700 - 900 μm. When loaded with irinotecan, it can decrease in size by up to 30%. The dose is 50 mg/ml, for a total dose of 100 mg per vial. The size of bead utilized in each treatment was at the treating physicians discretion.

Irinotecan loaded DEBIRI is delivered by trans-arterial chemoembolization (TACE). The primary function of the device is to embolize the arteries feeding the tumor site, causing tumor necrosis by starving it of nutrients and oxygen. The secondary function is to deliver irinotecan in a controlled manner. These functions combine to enhance the toxic effect of the drug on the tumor while minimizing systemic side effects.

All adverse events (AE) and serious adverse events (SAE) were recorded using the standards and terminology set forth by the Cancer Therapy Evaluation Program Common Terminology Criteria for Adverse Events, Version 3.0. Adverse events, defined as an untoward deviation in health away from baseline due to any cause, were recorded during the hospital stay and for 30 days following each treatment.

Follow-up assessments included a tri-phase CT scan of the liver within at least one to two months from the treatment. Evaluation of the enhancement pattern of the target lesion and tumor response rates were measured according to RECIST[16], EASL[17], and modified RECIST[18] criteria.

Data entry was monitored for completeness and accuracy at University of Louisville, and the data were queried when indicated. Source documents were requested and monitored for at least the first 5 patients from each site. A central assessment of tumor response was performed for all patients by the Principal Investigator at University of Louisville. When there was a discrepancy, the Registry PI and the site PI reexamined the data.

Once all the data were entered and all queries on data clarification forms resolved, the database was locked and the interim analysis performed. Data analysis was limited to descriptive reports of the number and characteristics of the patients treated and their clinical responses as well as their adverse events. Descriptive statistics were used to evaluate feasibility and safety. All demographic data have been incorporated into a summary that includes age, race, sex, height, weight, extent of liver disease, extent of hepatic failure, and CEA level. Descriptive statistics include the number and proportion of patients who completed planned therapy, the extent of hepatic and systemic toxicity, and, if the data allowed, the response to therapy.

All subjects have been evaluated for safety. Exposure to the study drug is summarized for all subjects. Summary statistics also include adverse events, hematology (white blood count, hemoglobin, and platelet count), and clinical chemistry (ALT, AST, total bilirubin, prothrombin time, and alkaline phosphatase). All toxicities were carefully monitored. Clinicopathologic data along with peri-operative complications were recorded. Analysis of data was done using JMP 4.0 and SPSS version 16.0.

## Results

Fifty-five patients with CRHM underwent 99 total treatments at the sites shown in Table 1. Forty were Caucasian, 9 African American, and 6 other. The median age of the patients was 62 years and the range was 34 to 82 years old, with more male (n = 34) than female (n = 21) (Table 2).

**Table 2:** Demographics based on treatment.

	**Variable**	**Total**
Gender	Male	34
	
	Female	21

Race	Caucasian	40
	
	African-American	9
	
	Other	6

Age (years)	N = 55	
	
	Mean	62
	Median	62
	± SD	10.6
	
	Range	34-82

Weight (kg)	N = 55	
	
	Mean	80.2
	Median	79.5
	± SD	17.6
	
	Range	45.4-127.7

Height (cm)	N = 55	
	
	Mean ±	148.5
	Median	147.4
	SD	9.3
	
	Range	132-173.8

Body Surface (m^2^)	N = 55	
	
	Mean ±	1.928
	Median	1.889
	SD	0.25
	
	Range	1.424-2.635

Twenty-eight patients had previously been treated with hepatic surgery, 54 with prior systemic chemotherapy, including FOLFOX in 35 patients, FOLFIRI in 15, Avastin in 37 patients, and other biologics in 9, with 2 patients receiving hepatic directed radiotherapy. The extent of liver involvement was 57% were <25%, 34% 26-50%, 9% were 51-75% Liver replacement.

Fifty-five total patients under went 99 irinotecan bead treatments, with most patients receiving 1 or 2 treatments based on the extent and location of the liver disease (table 3). If patients had unilobar disease then most underwent one treatment, if bilobar then two treatments. The extent of hepatic tumor burden was most commonly multifocal and involved the right lobe (Table 4). Extrahepatic disease was present in 25 patients, with the most common locations being lung (n = 15), bone (n = 2), lymph nodes (n = 5), and pelvis (n = 3). Median CEA levels at baseline prior to treatment was 26, (range 1.9 to 3533). Karnofsky status at baseline was prior to treatment 100-90 for most patients.

**Table 3:** MCC number of target lesions, size and location by CT.

	**Dose Administered**	**0-50 mg/m**^2^	**51-99 mg/m**^2^	**100 mg/m**^2^	**150 mg/m**^2^	**200 mg/m**^2^	**Total**
Number of target lesions	One	3	6	9	0	1	19
	
	One + Satellites	2	1	0	0	1	4
	
	Two	2	1	9	2	2	16
	
	Multinodular	6	10	18	2	8	44

Mean number of target lesions	N = 0 (missing) N = 83	13	18	36	4	12	83
	
	Mean ± Std	2.692	2.722	3.02	3	3.25	-
	
	Median	2	3	2.5	2.5	3	-
	
	Range	1-5	1-5	1-5	2-5	1-5	-

Anatomic site	RHL	7	14	18	2	5	46
	
	LHL	7	6	12	1	0	26
	
	Both	0	1	3	1	6	11

Diameter per tumour (cm)	N (tumors)	35	48	109	12	39	243
	
	Mean ± Std	2.58	2.59	3.17	4.125	3.9	-
	
	Median	1.8	2	2.4	2.15	3.4	-
	
	Range	1-10.1	0.5-7.4	1-14	1.1-9.5	1.5-12.2	-

Sum of diameter (cm)	N = 0 (missing) N = 83	13	18	36	4	12	83
	
	Mean ± Std	6.95	7.02	9-575	12.375	12.63	-
	
	Median	6.5	6.6	9.4	14.25	13.95	-
	
	Range	3.6-10.3	1-18.1	2-20	3.5-17.5	1.6-19.7	-

In 50% of patients, treatment was performed over two or more sessions (for example, where bilobar hepatic disease was treated). The level of embolization was lobar in 80% of treatments and segmental or subsegmental in 20%. A total dose of 100 mg of irinotecan was generally loaded into one DC/LC Bead vial (in most cases 100-300 microns size) (Table 4). In the majority of cases (n = 90), 100% of the loaded dose was administered for the first treatment and 80% of the dose for subsequent treatments. Complete occlusion was achieved in 28% of cases, near in 32%, and partial occlusion was achieved in 40%. The most common peri-procedural medications included opioids (100%), antiemetics (100%), steroids (44%), antihypertensive (82%), and intra-arterial lidocaine injection 2-4 cc prior to DC/LC Bead injection (55%). Antibiotic prophylaxis was at the physician's discretion and was used in 72% of patients.

**Table 4:** Details of Irinotecan DC/LC Bead Treatment.

**Variable**	**patients**	**N**
N of treatments = 99 Total # of patients = 55	One treatment	55(100%)
	
	Two treatments	28 (50%)
	
	Three treatments	12(9%)
	
	≥Four treatments	4(5%)

Maximum number of treatment sessions (for patients with bilobar disease) N (# of pts w/bilobar disease) 18	One session	2(11.1%)
	
	Two sessions	11(61.1%)
	
	Three, etc sessions	5(27.8%)

Irinotecan dose loaded per treatment	Mean = 110.17	
	median = 100	
	SD +/- 45	
	
	Range = 50-200	

Percentage of loaded volume per treatment	≤ 24 percent	2(2%)
	
	25-49	1(1%)
	
	50-74	13(13%)
	
	≥ 75	83(84%)

Irinotecan dose administered per treatment	0-50 mg	15(15%)
	
	51-99 mg	18(20%)
	
	100 mg	50(50%)
	
	150 mg	4(4%)
	
	200 mg	12(12%)

Degree of occlusion achieved per treatment	Complete	24(24%)
	
	Partial	40(40%)
	
	Near	35(35%)

A total of 14 Adverse Events were reported in 55 patients after the first treatment (Table 5). A statistically significant difference in the incidence of any adverse event was seen in patients who received greater that 100 mg versus patients who received 100 mg or less as their first treatment (p < 0.0001) The incidence of any adverse event during the first treatment was greater in those patients who received 100 mg or less than in those who received less than 100 mg (p < 0.0001) (Table 6). A total of 16 patients (29%) experienced 30 adverse events during the study period (Table 7). During the treatment cycles, no changes were seen in the liver chemistries or haematology parameters. The median hospital stay was 23 hours (range 23 hour to 13 days).

**Table 5:** Events after *1*^st ^*treatment *(based on dose received)

	**AE**	**# Occurrences**	**Event Rate %**
**50 mg dose** **N = 3**	Nausea	1	33.3
	
	Vomiting	1	33.3
	
	Hypertension	1	33.3
	
	Liver Dysfunction	1	33.3

			

**100 mg dose** **N = 45**	Nausea	2	4.4
	Vomiting	1	2.2
	
	Gastritis	1	2.2
	
	Pain	1	2.2
	
	Liver Dysfunction	1	2.2

			

**150 mg dose** **N = 1**	Nausea	1	100
	
	Vomiting	1	100
	
	Anorexia	1	100

			

**200 mg dose** **N = 6**	Liver dysfunction	1	16.7

**Table 6:** Incidence of Adverse Events by total dose administered (regardless of the interval of dosing).

**AE** **N = 30**	**<=100 mg dose**	**Event Rate %**	**<=200**	**Event Rate %**	**<=300**	**Event Rate %**
Nausea			2	6.7	3	10

Vomiting			2	6.7	3	10

HTN			1	3	3	10

Infection					1	3

Liver Dysfunction	1	3	2	6.7	3	10

Gastritis			1	3		

Dehydration			1	3		

Cholecystitis					1	3

Anemia			1	3		

Pneumonia			1	3		

Anorexia	2	6	1	3	1	3

**Table 7:** The type and incidence of adverse events by relation to bead treatment

**Adverse Event Description**	**# events**	**Adverse Event Grade**	**Adverse Event Outcome**	**Adverse Event Relationship**	**Adverse Event Explain**
Anorexia (n = 3 patients)	3	Grade 2	Resolved	Possibly Related	PE syndrome
	1	Grade 3	Resolved	Possible Related	

Hypertension (N = 1 patient)	4	1-2	Resolved	Not Related	Pre-existing condition

Liver dysfunction/failure (n = 4 patients)	3	1-2	Resolved	Possibly Related	Extent of Liver disease
	2	3	Ongoing	Possibly Related	
	1	5	Resolved	Possibly Related	

Nausea (n = 4 patients)	5	1-2	Resolved	Possibly Related	PE syndrome

Vomiting (n = 3 patients)	5	1-2	Resolved	Possibly Related	PE syndrome

Other: Gastritis, Dehydration, Anemia, Pneumonia (n = 4 patients) Cholecystitis (n = 1 patient)	5	1-2	Resolved	Possible Related	Chemotherapy
	1	3	Resolved	Possibly Related	Aberrant Infusion

During a median follow-up of 18 months, 12 patients died, with the most common cause being disease progression (Table 8). Only one patient died of an SAE that was judged to be an SAE possibly related to treatment. This 52 year-old male had a pre-operative bilirubin of 1.9 and an INR of 2.0. His liver disease included 4 lesions in segments 5-8, with the largest lesion measuring 4.2 cm and a total liver involvement of 26-50%. The total target lesion size measured 12.9 cm. He also had extrahepatic disease involving the pancreas, spleen, and lung. Treatment was delivered to the right lobe and consisted of 2 vials of DEBIRI loaded with 200 mg of Irinotecan. One vial contained beads measuring 300-500 μm and the other contained beads measuring 300-500 μm. Zofran was given during the procedure, ciprofloxacin and flaygl were given afterward, and pain was managed with an epidural. Following the procedure, the patient had a 3-day hospital stay for nausea and was discharged home without incident. When the patient returned complaining of nausea 28 days after the procedure, he was diagnosed with liver dysfunction, and died of this disorder 30 days later.

**Table 8:** Disposition of patients as per follow-up.

**Screened**	**3 month**	**6 month**	**12 months**	**18 month**
55	55	53	46	26

**Reason for Death**			**n (% from total n)**	

Disease Progression			**12 (22)**	

SAE			**1 (2)**	

**Figure 1:**
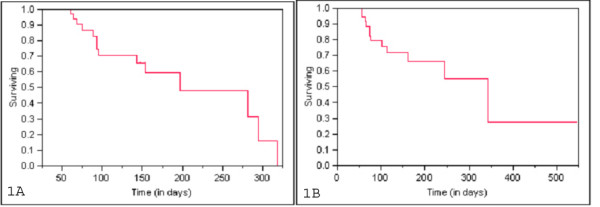
a) Disease Free Survival of patients treated with Irinotecan drug eluting beads with liver dominant hepatic metastasis after failing standard chemotherapy b) Overall Survival of patients treated with Irinotecan drug eluting beads with liver dominant hepatic metastasis after failing standard chemotherapy

When treatment response was measured by the EASL criteria, we had an observed response (defined as CR, PR, and SD) in 89% of patients at 3 months, 80% at 6 months, and 54% at 12 months (Table 9). When treatment response was judged by the RECIST criteria, 71% responded at 3 months, 56% at 6 months, and 40% at 12 months, while 9 patients suffered progression in their liver disease during follow up (Table 10). When the end-point was any progression, either in the liver or elsewhere in the body, the mean disease-free survival time was 206.09 days and the median disease-free survival time was 197 days (Figure 1). The median overall survival from the time of first treatment was 247 days and the median was 343 days (Figure 1). Six patients (10%) were downstaged from their original disease status. Of these, four were treated with surgery and two with RFA.

**Table 9:** EASL Tumour response.

**Tumour Response (N = 55)**	**3 month**	**6 month**	**12 months**	**18 month**
Complete Response	3	3	2	1

Partial Response	18	16	17	16

Stable Disease	28	25	11	8

Progressive Disease	5	2	4	1

Deaths	1	7	13	NA*

Not Reached Follow	0	2	7	29

Lost to Follow Up	0	0	2	0

Total	55	55	55	55

*Data not available at time of report but including at least 13 deaths

**Table 10:** RECIST Tumour response.

**Tumour Response (N = 55)**	**3 month**	**6 month**	**12 months**	**18 month**
Complete Response	2	2	1	1

Partial Response	4	2	2	2

Stable Disease	33	27	19	22

Progressive Disease	15	15	12	1

Deaths	1	7	13	NA*

Not Reached Follow	0	2	7	29

Lost to Follow Up	0	0	2	0

Total	55	55	55	55

*Data not available at time of report but including at least 13 deaths

Predictors of overall survival from the time of first bead treatment were evaluated in an attempt to identify factors that predicted outcome. Neither number of liver lesions, size of liver lesions or extent of liver replacement (<= 25% vs >25%) were predictors of overall survival. Only the presence of extrahepatic disease (p = 0,001), extent of prior chemotherapy (failed 1^st ^and 2^nd ^liver vs > 2 line failure) (p = 0,007) were predictors of overall survival in multivariate analysis.

## Discussion

This interim report includes data from five US based sites and six European sites. All patients had unresectable hepatic metastases from colorectal cancer and were treated with at least one injection of Irinotecan-loaded DEBIRI at dosages that ranged from 50 mg to 200 mg per treatment.

When chemoembolization was first used to treat metastatic colorectal cancer, the agent was a mixture of ethylcellulose microcapsules and mitomycin C supplemented with gelatin sponge[11]. Since then, a range of embolization devices and ancillary drug regimens have been employed [19-23]. The patient populations have varied among the published studies, and because of this, caution should be used when evaluating the results. In a recent review, Vogl et al. report median survivals that range from 9 to 62 months and morphological responses that vary from 14 to 76%[24].

In one of the largest series reported to date, Vogel et al evaluated the efficacy of TACE for improving survival and achieving local control in patients with liver metastases from colorectal cancer[24]. Two hundred and seven patients with liver metastases from colorectal cancer were treated with repeated TACE in at 4-week intervals. A total of 1,307 chemoembolizations were performed, with a mean of 6.3 sessions per patient. The average age of the 207 patients was 68.8 years (range, 39.4-83.5 years). Of these, 158 were treated for palliation, 35 to reduce symptoms, and 14 as adjuvant therapy. The chemotherapy consisted of mitomycin C with or without gemcitabine, and embolization was performed with lipiodol and starch microspheres to achieve vessel occlusion. Local control measured by the RECIST criteria were as follows: partial response in 12% of patients, stable disease in 51% and progressive disease in 37%. The 1-year survival rate after TACE was 62%, but the 2-year survival rate was reduced to 38%. The median survival time from the date of diagnosis of metastases was 3.4 years the median survival time from the start of TACE treatment was 1.34 years. The median survival time of the palliative group was 1.4 years, of the symptomatic group 0.8 years and of the neoadjuvant group 1.5 years. Vogl *et al*, concluded that TACE is an effective minimally-invasive therapy for neoadjuvant, symptomatic or palliative treatment of liver metastases in colorectal cancer patients[24]. The results presented here are comparable to Vogl's and they were achieved with significantly fewer treatments (two versus six).

In spite of these promising results from a number of studies, none have demonstrated a significant improvement in survival after chemoembolization[22]. observation, plus the need for a more careful cost-benefit analysis, suggests that additional prospective randomized trials should be done [25-27]. The fact that substantial extrahepatic progression is often observed after regional treatment for liver metastases further suggests that systemic chemotherapy should be added to chemoembolization[28,29].

In this study, all the patients did not receive the same adjunct medication or the same type of treatments with the Irinotecan drug eluting device. Thus our data cannot be used to establish specific medical protocols for the US or Europe.

The same number of patients received one treatment versus multiple treatments, while the vast majority of patients received the planned pre-treatment loaded dose based on vascularity as well as tumor distribution. There was a 100% technical success in the use of the device, and there were no significant serious adverse events related to its insertion.

The safety of this device is shown by the fact that only one patient suffered a serious adverse event. This patient had an especially large tumor burden, which required a high dose of irinotecan (200 mg). The remaining adverse event profile is consistent with other well established as well as historical hepatic arterial therapy treatments.

We observed a post-embolic syndrome (characterized by nausea, vomiting, dehydration and pain) in patients who received multiple treatments, with a cumulative dose of 300 mg or greater. However, none of these patients suffered any serious adverse events associated with the treatment. The clinical and laboratory evaluation showed no significant variations in lab values that could be attributed to treatment.

Overall, we observed a very strong response rate at three months and a durable response at both six and twelve months in those patients that were measurable by EASL criteria. Not surprisingly, the response rates were reduced when measured by the traditional RECIST criteria, because of its well-documented limitations[30].

The results of this study, when measured by time-to-progression and overall survival, represent a remarkable achievement, given that most of the patients had already been treated for their metastatic disease, some with as many as three or four agents.

## Conclusion

In conclusion, this interim report demonstrates that the Irinotecan loaded DEBIRI is safe and effective in patients with unresectable metastatic colorectal cancer. This treatment shows a significant benefit for patients who have failed first and second line therapy and is potentially an effective therapy when compared to the historical response rates to third and fourth line systemic chemotherapy.

## Competing interests

RCGM: Consultant for Biocompatibles.

## Authors' contributions

RCGM have made substantial contributions to conception and design, or acquisition of data, or analysis and interpretation of data, involved in drafting the manuscript or revising it critically for important intellectual content. KR have made substantial contributions to conception and design, or acquisition of data, or analysis and interpretation of data, involved in drafting the manuscript or revising it critically for important intellectual content. DT have made substantial contributions to conception and design, or acquisition of data, or analysis and interpretation of data, involved in drafting the manuscript or revising it critically for important intellectual content. RO'H have made substantial contributions to conception and design, or acquisition of data, or analysis and interpretation of data, involved in drafting the manuscript or revising it critically for important intellectual content. PB have made substantial contributions to conception and design, or acquisition of data, or analysis and interpretation of data, involved in drafting the manuscript or revising it critically for important intellectual content. RP have been involved in drafting the manuscript or revising it critically for important intellectual content. MR have been involved in drafting the manuscript or revising it critically for important intellectual content. FS have been involved in drafting the manuscript or revising it critically for important intellectual content. AS have been involved in drafting the manuscript or revising it critically for important intellectual content. CT have been involved in drafting the manuscript or revising it critically for important intellectual content. All authors have seen and approved final version to be published.
